# Chemical Components, Antioxidant Activity, and Glycemic Response Values of Purple Sweet Potato Products

**DOI:** 10.1155/2022/7708172

**Published:** 2022-04-15

**Authors:** Siti Nurdjanah, Samsu Udayana Nurdin, Sussi Astuti, Venni Elsa Manik

**Affiliations:** Department of Agriculture Product Technology, Faculty of Agriculture, Lampung University, Indonesia

## Abstract

Purple sweet potato is a source of starch with high potential to be developed as a functional food. It can be boiled and consumed as a snack or processed into intermediate goods such as flour. The flour can then be modified through partial gelatinization and followed by a retrogradation process to produce resistant starch-rich purple sweet potato flour. The study was aimed at obtaining the glycemic response values of purple sweet potato products, namely, boiled purple sweet potatoes (BSP), purple sweet potato noodles (SPN), and resistant starch-rich purple sweet potato noodles (RSPN). SPN was prepared from conventional purple sweet potato flour, whereas RSPN was made from resistant starch-rich purple sweet potato flour. Moreover, water, ash, protein, fat, and carbohydrate; total phenolic, anthocyanin, and resistant starch contents; and the rate of hydrolysis of starch were evaluated. Ten subjects were involved in the estimation of glycemic response determined by the area under the curve (AUC) of the blood glucose after consuming products. Glucose syrup was used as a reference. The glycemic response data were processed using ANOVA and further tested using LSD at *p* < 0.05. The results showed RSPN had the lowest glycemic index value if compared to BSP and SPN (58.7, 63.5, and 83.7) and fell under food with medium GI, but the differences were not statistically significant. RSPN and SPN were classified as medium GI; however, the process of partial gelatinization followed by retrogradation during the preparation of flour used for raw material in making RSPN has successfully maintained the total phenol and anthocyanin and increased resistant starch content of the noodle Processing of purple sweet potato flour into noodle lowered the GI category, and when the flour was partially gelatinized and retrograded, the noodle had more potentiality as a functional food due to their high total phenolic and anthocyanin content.

## 1. Introduction

Purple sweet potato is a food source rich in carbohydrates, primarily starch. As a starch source, purple sweet potatoes have a high potential to be developed as a functional food due to its bioactive components that are said to be good for health. One of the bioactive components with physiological functions is anthocyanins [1]. Anthocyanins in purple sweet potatoes act as an antioxidant [2], anticancer, antihyperglycemic, and antihypertension [2, 3]. Boiled, steamed, or fried purple sweet potatoes can be consumed as a staple food or a snack. However, with high water content (60%-80%) [4], they have low storability after harvest. To lengthen the shelf life of purple sweet potatoes, processing them into flour is an appropriate alternative.

Processing purple sweet potatoes into flour has other benefits such as having more flexible utilization, saving storage space, and being available anytime without waiting for the harvest season. Another benefit is that it is easy to modify purple sweet potato flour to maintain its bioactive components and improve its physiological functions. Nurdjanah [5] reported that partial gelatinization by heating at 90°C for 15-30 mins could maintain the anthocyanin level in purple sweet potato flour. When this partial gelatinization was followed by a cooling process at 5°C for 48 hours, retrogradation happened, and the result was resistant starch-rich purple sweet potato flour that highly inhibited *α*-glucosidase enzyme [6] and *α*-amylase enzyme activities [7].

Research by Sajilata et al. [8] states that resistant starch has a hypoglycemic effect because the hydrolysis of resistant starch by digestive enzymes needs a long time and glucose is released slowly. Consuming food with resistant starch effectively lowers the postprandial blood glucose level, increases insulin sensitivity [9], and regulates insulin resistance for patients with diabetes mellitus [9]. The lowering of the blood glucose level starting from 1 hour postprandial is due to the positive physiological effects of consuming resistant starch. These physiological effects happen in two mechanisms. First, *α*-amylase enzyme activity in the small intestines is inhibited, slowing down glucose digestion [10]. Second, resistant starch produces short-chain fatty acids (SCFA), primarily propionate acid, which increase the secretion and sensitivity of insulin in adipose tissues [11].

Research regarding the glycemic response of processed food made from high-carbohydrate tubers such as purple sweet potatoes is still limited. The same ingredient has different glycemic indexes when processed differently because the characteristics and physiochemical properties have changed [12]. For example, different treatments can cause an ingredient to be more easily digested and absorbed thus causing rapid blood sugar level increases or changing the starch into resistant starch. The glycemic response of purple sweet potato products such as boiled purple sweet potatoes, noodles, and resistant starch-rich noodles is still mostly unknown. Thus, this research focused on evaluating the glycemic response and predicting the glycemic index of several purple sweet potato products. Processed product purple sweet potatoes in the form of boiled and two types of noodles were chosen because boiled sweet potatoes are commonly eaten as a snack food whereas noodles are a favourite food and even become a substitute for staple food after rice. BPS [13] reports that in Indonesia, the consumption rate of noodles is 80 grams/capita/week. This high consumption causes Indonesia to be ranked 2nd as the largest noodle-consuming country after China [14]. This opens up opportunities to develop noodles from raw materials other than wheat, which is still imported. One of the substitutes which are prospective as a raw material for noodles is purple sweet potato flour.

## 2. Materials and Methods

### 2.1. Materials and Equipment

The main material of this research was local purple sweet potatoes from a traditional market in Way Kandis, Bandar Lampung, Indonesia. Chemical substances for analysis included NaOH, HgO, K_2_SO_4_, H_2_SO_4_, 95% alcohol, 0.02 N HCL, H_3_BO_3_, Na_2_CO_3_, gallic acid, 0.2% citric acid, KCl buffer solution, pH 4.75 sodium acetate buffer solution, pH 1.5 KCl-HCl buffer solution, phenol, KOH 4 M, pepsin, glucose, *α*-amylase enzyme from *Aspergillus oryzae* (powder), 3,5-dinitrosalicylic acid (DNS), and glucoamylase enzyme.

Equipment used in this study was a modified rotary drum dryer, refrigerator, peeler, and cabinet dryer. Equipment used for analysis included Kjeldahl flask, boiling flask, Soxhlet extractor, vortex, centrifuge spectrophotometer Genesys S10-UV-VIS, water bath, and blood glucose testing equipment Accu-Chek Performa.

### 2.2. Sample Preparation

#### 2.2.1. Boiled Purple Sweet Potatoes

Fresh purple sweet potatoes (1 kg) were sorted, washed, drained, and boiled in boiling water (2 L) for about 30 minutes. Boiled purple sweet potatoes were then taken out, drained, peeled, and cut into 2 × 2 × 2 cm cubes to be served to respondents.

#### 2.2.2. Purple Sweet Potato Noodles

Purple sweet potatoes were cleaned and grated to 1 mm thickness. Purple sweet potatoes were then dehydrated in a cabinet dryer at 60°C for about 16 hours until the water content was about 10%. Dehydrated purple sweet potatoes were processed into flour using a hammer mill and sieved through the 80-mesh sieve.

Purple sweet potato flour noodles were prepared following the method of Yuliana et al. [15] with slight modification. Flour was weighed to 200 g, put into the mixing bowl along with carrageenan (1% of purple sweet potato flour) and water (1 : 1 ratio of water to flour), and kneaded until a homogenized dough was formed. The dough was then steamed for 3 minutes at 100°C and put through a sheeter to form sheets then a noodle maker to form noodle strands. The noodle strands were put into small cups and dehydrated in a cabinet dryer at 60°C for 12 hours to make dried noodles. Dried noodles were soaked in 1 : 1 water for 1 minute before steaming for 10 minutes.

#### 2.2.3. Resistant Starch-Rich Purple Sweet Potato Noodles

Purple sweet potatoes were cleaned, grated, and heated to 90°C for 30 minutes in a rotary drum dryer. For the cooling process, purple sweet potatoes were then cooled at 25°C for 1 hour and put into the refrigerator at 5°C for 48 hours. Cold purple sweet potatoes were dried in a cabinet dryer at 60°C for about 16 hours until the water content reached 10%, then processed into flour using a hammer mill and sieved through the 80-mesh sieve [16]. The process to make the noodle was the same as the purple sweet potato noodles explained previously.

### 2.3. Determination of Proximate Analysis

The proximate analysis of water, ash, protein, fat, and carbohydrate by difference contents followed the AOAC (2005) method [17].

### 2.4. Total Phenolic Content

Total phenolic content analysis used a modified version of the method of Nurdjanah et al. [5]. The extract sample was measured to 0.2 mL and mixed with 0.2 mL of distilled water and 0.2 mL of Folin-Ciocalteu reagent in a vortex for 1 minute. Then, 4 mL of 2% sodium carbonate (Na_2_CO_3_) was added before mixing again in a vortex for 1 minute and left in a dark room for 30 minutes at room temperature. The absorbance of gallic acid reaction with the Folin-Ciocalteu reagent was then read at 760 nm wavelength. A blank form with the same procedure as the sample was prepared. The absorbance results were plotted on a standard curve for gallic acid using the linear regression equation with gallic acid concentration on the *x* axis and absorbance on the *y* axis. A standard curve for gallic acid was made by mixing 1 mg of gallic acid with distilled water (until the total volume was 100 mL), preparing a series of 0%, 20%, 40%, 60%, 80%, and 100% dilution from that gallic acid solution, and giving them the same treatment as the sample. The results were shown in the standard curve equation:

(1) $$\begin{array}{c} y=ax+b. \end{array}$$

### 2.5. Total Anthocyanin Content

The total anthocyanin concentration was measured using the method by Giusti and Wrolstad [18]. Two sample solutions were prepared. The first one was a solution for pH 1.0 using KCl buffer solution, and the second one was for pH 4.5 using Na acetate buffer solution. Each buffer solution was added with 1 mL purple sweet potato extract until the total volume was 10 mL (dilution factor = 10). The absorbance of each diluted sample was measured at 500 nm and 700 nm wavelengths using the following equation:

(2) $$\begin{array}{c} A={\left(A_{\lambda \text{ vis }\max}-A_{700}\right)}_{\text{pH }1.0}-{\left(A_{\lambda \text{ vis }\max}-A_{700}\right)}_{\text{pH }4.5}. \end{array}$$

The total anthocyanin content was determined using the following equation:

(3) $$\begin{array}{c} \text{total anthocyanin content }\left(\text{mg}/\text{L}\right)=\frac{A\times \text{MW}\times \text{DF}\times 1000}{\varepsilon \times 1}, \end{array}$$

where *A* is absorbance, MW is molecular weight of cyanidin 3-glucoside (449), DF is dilution factor, *ε* is coefficient of cyanidin 3-glucoside molar extinction (26,900 L/cm), and 1 is cuvette thickness.

### 2.6. Resistant Starch Content

The resistant starch content was determined following the Goni et al. [19] method with a small modification in determining the glucose level. Into a centrifuge tube, 100 mg of the sample and 10 mL of 1.5 pH KCl-HCl buffer solution were added. Then, the pH adjustment to pH 1.5 was done by adding HCl (2 M) or NaOH (0.5 M), and 2 mL of pepsin solution was added (1 g pepsin per 10 mL KCl-HCl buffer solution).

The mixture was put into a 40°C water bath for 60 minutes, then cooled at room temperature. pH was adjusted to pH 6.9 by adding NaOH (0.5 M), and 1 mL of *α*-amylase enzyme solution was added. The mixture was then incubated for 16 hours in a water bath at 37°C with constant stirring and centrifuged for 15 minutes at 3000 rpm. The resulting supernatant was thrown away, and 10 mL of distilled water was added to the residue to be centrifuged again (15 minutes, 3000 rpm). The supernatant was then separated. The residue was mixed together with an additional 3 mL of distilled water and added with KOH (4 M) before being incubated in a Memmert shaker water bath for 30 minutes at 25°C with constant stirring. Then, 5.5 mL HCl (2 M) and 3 mL sodium acetate (0.4 M) buffer solutions were added, and pH was adjusted to 4.75 by adding HCl (2 M).

After that, 80 *μ*L of glucoamylase enzyme was added and mixed in and left in a Memmert water bath for 45 minutes at 60°C. The supernatant was separated using a centrifuge tube (15 minutes, 3000 rpm) and kept while 10 mL of distilled water was added to the residue and centrifuged again. The resulting residue was then thrown away. The supernatant was mixed with the previously obtained supernatant, and distilled water was added to reach 50 mL in volume. Glucose content was determined using the phenol-sulfuric acid method, and resistant starch content was determined by multiplying the glucose content by 0.9.

### 2.7. Rate of Hydrolysis with *α*-Amylase Enzyme

The hydrolysis rate of starch with *α*-amylase enzyme was determined following the modified method by Nurdjanah et al. [5]. Powdered purple sweet potato noodle was measured to 1 g and added into a reaction tube with 9 mL of distilled water, heated to 90°C for 15 minutes in a water bath, and cooled. The sample was then added with 1 mL of *α*-amylase enzyme and 3 mL of pH 7 phosphate (0.1 M) buffer solution; incubated at 37°C for 0, 60, and 120 minutes; and centrifuged at 3000 rpm for 15 minutes. The glucose content of the resulting sample was then determined using the phenol-sulfuric acid method.

### 2.8. Glycemic Response

The glycemic response was evaluated in several steps, which were (a) ethical clearance application, (b) subject candidate recruitment, (c) subject candidate selection, (d) research explanation and informed consent, and (e) glycemic response evaluation. This research was done after obtaining ethical clearance from the authorized committee of ethics. The recruitment and selection of subject candidates were done using the purposive sample method that fulfilled the inclusive and exclusive criteria. The inclusive criteria were subjects aged between 18 and 30 years of age of any gender with normal BMI (18.5-22.9 kg/m^2^) and in healthy conditions. The exclusion criteria included having no history of diabetes, no current digestive issues, not being in the middle of treatment, not using illegal drugs, not smoking, and not drinking alcohol. There were 10 candidates selected as respondents, and they were given a short explanation of this research before signing informed consent forms.

The glycemic response was determined using a modified method of El [20]. Ten respondents were asked to fast (only drinking water was allowed) for about 10 hours from 8pm the previous night until the following morning. Testing was done at 8am by giving the respondents 50 g of carbohydrates from each purple sweet potato products and pure glucose. The glycemic response of respondents was tested every three days for two weeks.

In the first week, respondents were given 50 g of pure glucose dissolved in 200 mL water as comparison. Respondents consumed the pure glucose solution for 5-10 minutes, and their glycemic responses were analyzed. The next test was done three days later when respondents were given boiled purple sweet potatoes, and their glycemic responses were analyzed. The following week, the process was repeated with purple sweet potato noodles and again in the final week with resistant starch-rich purple sweet potato noodles.

After consuming each purple sweet potato products, 0.6 *μ*L blood samples were obtained from respondents using the finger-prick capillary blood sample method at minutes 0, 30, 60, 90, and 120. Blood samples were taken while respondents were in a relaxed condition. In the graph, *x* axis was for time in minutes and *y* axis was for the blood glucose level. The area under the curve (AUC) was then calculated with the following equation:

(4) $$\begin{array}{c} \text{AUC}=\int_{0}^{120}f\left(x\right)dx. \end{array}$$

### 2.9. Statistical Analysis

This research was arranged in a completely randomized design with three replications. The data of proximate composition were reported for their means and standard deviation, whereas total phenol, total anthocyanin, and resistant starch content' data were then analyzed for their homogeneity and additivity using the Bartlett and Tukey tests, then processed using analysis of variance (ANOVA), and further tested using the least significant difference (LSD) test. All the cooked purple sweet potato products and pure glucose were then evaluated for their glycemic responses following the method of El [20] with ten respondents. The results of the blood glucose response evaluation of each subject were displayed on *x* axis (time) and *y* axis (glycemic response). The glycemic responses of ten respondents on each product were averaged to reveal the curve chart. The effect of consuming each product on the glycemic responses was analyzed for its normality, followed by the paired samples test using the software SPSS 16.0.

## 3. Results and Discussion

### 3.1. Proximate Analysis

In this research, proximate analysis was completed to figure out the nutritional content of these products such as water, fat, ash, and carbohydrate by difference. One factor that affects blood glucose response is the composition of nutrition [21]. Boiled purple sweet potatoes, purple sweet potato noodles, and resistant starch-rich purple sweet potato noodles all had different proximate compositions due to their different processes. Our results were comparable with those previously reported by Hossain [22] and Darwish et al. [23]. The result of the proximate analysis can be seen in Table 1.

### 3.2. Total Phenolic, Anthocyanin, and Resistant Starch Contents

The results of total phenolic, anthocyanin, and resistant starch contents are presented in Table 2.

#### 3.2.1. Total Phenolic Content

This research revealed that the highest total phenolic content of 357.33 mg GAE/100 g was found in boiled purple sweet potatoes (Table 2). This might be caused by the heat treatment that inactivated phenolase enzymes through the process of steaming, boiling, or roasting (Mahmudatussa'adah et al. [24]). Resistant starch-rich purple sweet potato noodles contained 327.10 mg GAE/100 g of total phenolic content while the regular purple sweet potato noodles contained only 195.58 mg GAE/100 g of total phenolic content. This smaller amount in purple sweet potato noodles might be due to its processing.

In this research, the total phenolic content of resistant starch-rich purple sweet potato noodles was lower than that of boiled purple sweet potatoes because in the noodle-making process, there was direct heat contact during dough steaming before making noodle sheets and dry noodle steaming. When boiling purple sweet potatoes, the skin stayed on thus avoiding direct heat contact between the water and the purple sweet potato flesh. According to Padda and Picha [25], the process and temperature of making purple sweet potato products affect the total phenolic content significantly. The higher the temperature and the longer the heating process, the lower the total phenolic content in the purple sweet potato product.

#### 3.2.2. Total Anthocyanin Content

Boiled purple sweet potatoes showed the highest amount of total anthocyanin content with 95.64 mg/100 g (Table 2). Meanwhile, resistant starch-rich purple sweet potato noodles and regular purple sweet potato noodles had 93.94 mg/100 g and 44.71 mg/100 g of total anthocyanin content, respectively. The high amount of total anthocyanin content in boiled purple sweet potatoes in this research was because they were boiled with skin on, causing only a very little amount of anthocyanin dissolved in the boiling water [26].

The higher amount of total anthocyanin content in boiled purple sweet potatoes than in other products was directly proportional to the amount of total phenolic content. Heat treatment on purple sweet potatoes is predicted to inactivate the enzymes that caused browning such as polyphenol oxidase and peroxidase [27], thus increasing the concentration of monomeric anthocyanin. Truong et al. [28] state that cooking by heating them for 25 minutes increases the concentration of monomeric anthocyanin in some purple sweet potato cultivars.

Both SPN and RSPN showed lower anthocyanin content when compared to boiled purple sweet potato anthocyanin content. This was probably because during the thermal process anthocyanins experience deglycosylation, nucleophilic attack of water, cleavage, and polymerization [29]. These reactions eventually have led the total anthocyanin content to decrease. Further results showed that SPN failed to maintain anthocyanin content because the raw material for SPN was conventional purple sweet potato flour. Conventional purple sweet potato flour was made by slicing sweet potatoes and then drying at 50°C for about 12 hours until the moisture content reached 10% without any blanching treatment. Drying without blanching may cause anthocyanin degradation due to activation of some enzymes. Some enzymes responsible for anthocyanin degradation are peroxidase enzyme [30], polyphenol oxidase, and catecholase [31]. It was also found that RSPN showed higher retention of anthocyanin content. RSPN was prepared from purple sweet potato flour that has undergone partial gelatinization and retrogradation before drying. The partial gelatinization process involved heating at 90°C for 15 minutes. This condition has caused anthocyanin-degrading enzymes to become inactive and thus prevented anthocyanin from severe degradation.

#### 3.2.3. Resistant Starch Content

The result of this research revealed that boiled purple sweet potatoes had the lowest amount of resistant starch with only 2.99% (Table 2). This might be caused by the cooking process, primarily boiling. The cooking process increases the amount of hydrolyzed starch due to complete starch gelatinization making it more vulnerable to enzyme attacks and easily digestible [32]. The resistant starch content in purple sweet potato noodles was only 6.52%, albeit still higher than in boiled purple sweet potatoes. The low amount of resistant starch in the purple sweet potato noodles was caused by the gelatinization process during the making of noodle sheets from dough and the steaming of noodles.

The highest amount of resistant starch (14.29%) was found in resistant starch-rich sweet potato noodles. This relatively high amount was because of the partial gelatinization of purple sweet potato flour at 90°C for 30 minutes and the following retrogradation at 5°C for 48 hours. Nurdjanah and Yuliana [16] stated that that process could result in purple sweet potato flour with 31.89% of resistant starch content. The decrease from that amount happened in the noodle-making process, specifically repeated heat treatments from creating the noodle strands until the noodles were ready to serve.

### 3.3. Rate of Starch Hydrolysis with *α*-Amylase Enzyme

**Figure 1:**
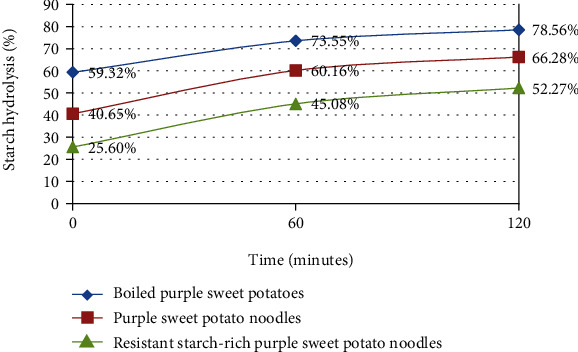
The rate of starch hydrolysis of purple sweet potato processed products.

The highest hydrolysis rate in 120 minutes happened in boiled purple sweet potatoes with 78.56% ± 0.26%. Meanwhile, the hydrolysis rate of purple sweet potato noodles reached 66.28% ± 1.88% with the lowest number of 52.27% ± 1.40% belonging to resistant starch-rich sweet potato noodles. The rate of hydrolysis with *α*-amylase enzyme was inversely proportional to the resistant starch content of purple sweet potato products. High resistant starch content in food may decrease the enzyme hydrolysis rate [10]. Resistant starch-rich purple sweet potato noodles had the lowest rate of enzyme hydrolysis because this product was made from partially gelatinized and retrograded purple sweet potato flour with high resistant starch content, primarily type 3. In the cooling process of the ingredient, there was recrystallization of dissolved amylose polymer chains, causing the partially gelatinized starch to reassociate and form a double helix and be stabilized by hydrogen bonds [32]. That entire process resulted in starch that was not easily digestible with amylase enzyme [33].  Figure 1 represents the rate of starch hydrolysis of purple sweet potato processed products.

### 3.4. Glycemic Response Values

#### 3.4.1. Characteristics of Respondent Subjects

This research was completed after obtaining ethical clearance from the Research Ethics Committee Faculty of Medicine University of Lampung Number 2985/UN26.8/DL/2017. Respondent subject recruitment was conducted through socialization with several students from the Agricultural Technology Department University of Lampung and interviewing them regarding their health history. The weight, height, and BMI of candidates were then observed. Candidates selected to be respondent subjects must fulfill the inclusive and exclusive criteria. The inclusive criteria were subjects aged between 18 and 30 of any gender with normal BMI (18.5-22.9 kg/m^2^) and in healthy conditions. The exclusive criteria included having no history of diabetes, no current digestive issues, not being in the middle of treatment, not using illegal drugs, not smoking, and not drinking alcohol. There was a total of 10 respondent subjects selected.

Respondent subjects were given an explanation of the research and observation that would be done before voluntarily signing the informed consent forms. All respondent subjects reserved the right to quit if they felt harmed or aggrieved. There were 7 female and 3 male respondent subjects in this research. The more respondent subjects the better [34]. However, this amount was deemed good enough for this research. Age, weight, and height of respondent subjects were obtained to know the characteristics of respondent subjects, as shown in Table 3.

The average age of respondent subjects was 22 years with 53.5 kg weight, 1.6 m height, and 20.83 kg/m^2^ BMI. This information indicated respondent subjects' good nutritional intake with normal BMI. According to *WHO: Asia Pacific Perspective* in the PERKENI (Indonesian Endocrinology Society) consensus, the normal BMI for Indonesians was between 18.5 and 22.9 kg/m^2^. The preprandial blood glucose level was obtained by averaging the four tests done at minute 0, indicating no diabetes history with the numbers all under 100 mg/dL.

Blood samples were obtained using lancet pens on respondent subjects' pointer, middle, and ring fingers. The respondent subjects' fingers were first cleaned and sterilized with an alcohol swab. For this research, the volume of the blood sample needed was minimum 0.6 *μ*L. The blood glucose level was measured using the Accu-Chek Performa digital glucometer. The blood samples obtained were touched onto the censor crack at the end of the test strip, and the results were read on the screen.

#### 3.4.2. Intervention on Respondent Subjects

Respondent subjects received food intervention of pure glucose syrup as a comparison in addition to the purple sweet potato products of boiled purple sweet potatoes, purple sweet potato noodles, and resistant starch-rich purple sweet potato noodles. Each food intervention was given 3–4-day intervals for recovery. Respondent subjects fasted for around 10 hours and were tested for their preprandial blood glucose levels. The testing was done in a comfortable room and relaxed condition with no influence from physical activities.

The first food consumed was 50 g of pure glucose syrup dissolved in 200 mL of water for control. Pure glucose syrup was the recommended item to act as control when determining the glycemic index values of other foods due to its standardized and uniform composition, unlike white bread. Respondent subjects needed 3 minutes to consume the glucose syrup. Blood samples were taken for blood glucose level testing at minutes 30, 60, 90, and 120 afterwards. In order, food interventions given after pure glucose syrup were boiled purple sweet potatoes, purple sweet potato noodles, and resistant starch-rich purple sweet potato noodles. The serving size for each product was equivalent to 50 g of carbohydrate by difference, as shown in Table 4.

The biggest serving size was for boiled purple sweet potatoes, and the smallest was for resistant starch-rich purple sweet potato noodles. This research did not consider soluble and insoluble fiber; thus, the available carbohydrate content could not be measured. The serving size in this research was determined using the carbohydrate by the difference method without revealing the total available carbohydrate to be digested by the body. The serving size of resistant starch-rich purple sweet potato noodles should have been bigger because resistant starch was not easily digested, lowering the total available carbohydrate.

Boiled purple sweet potatoes were served on the second testing (day 4) with 145 g serving size per person. Respondent subjects needed about 10 minutes to consume the sample. Purple sweet potato noodles were served on the third testing (day 8) with 112.87 g serving size per person and resistant starch-rich purple sweet potato noodles on the fourth testing (day 12) with 112.87 g per person. Respondent subjects needed about 15 minutes to consume these samples, which was quite a long time, due to the less desirable taste (distinctively sweet potato-like) and the portion size that was bigger than the regular noodle soup dishes. The amount of time it took to consume these products might affect the blood glucose level results because products consumed earlier would be digested earlier, too.

#### 3.4.3. Glycemic Response Evaluation

**Figure 2:**
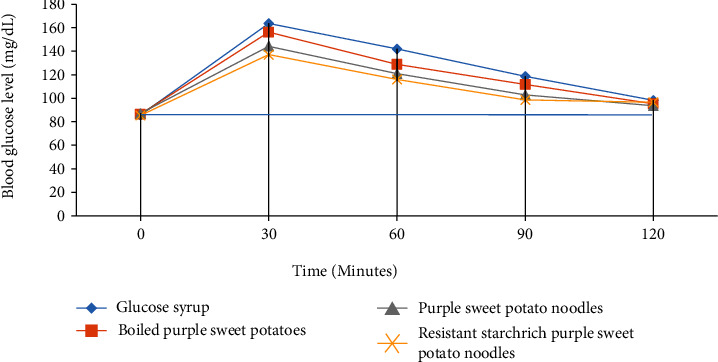
The curve of the glycemic response of pure glucose syrup and purple sweet potato products.

The glycemic response was determined from how respondent subjects' blood glucose levels responded to the samples of pure glucose syrup, boiled purple sweet potatoes, purple sweet potato noodles, and resistant starch-rich purple sweet potato noodles. The data from respondent subjects' blood glucose tests were entered with time on the *x* axis and blood glucose level on the *y* axis, and a curve was formed as seen in Figure 2.

Figure 2 shows that the peak of blood glucose from all four samples happened at minute 30 with pure glucose syrup reaching the highest level. It was followed by boiled purple sweet potatoes, purple sweet potato noodles, and resistant starch-rich purple sweet potato noodles consecutively.

The peak of blood glucose increment happens around 15-45 minutes after consuming food, depending on how fast the body digests and absorbs carbohydrates. The glycemic response of food products is shown by the fluctuation curve of glucose absorption in the blood after consuming them. Food components that are absorbed quickly by the body will cause high fluctuation in blood glucose levels in a short time. The curve with the lowest peak belongs to resistant starch-rich purple sweet potato noodles, indicating the slow digestion process that causes the slow release of blood glucose until 2 hours postprandial.

**Figure 3:**
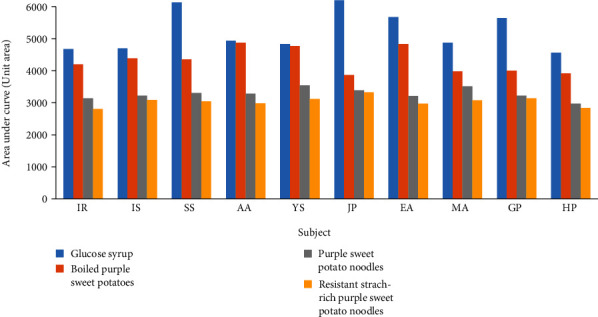
The area under the curve of each respondent subject for pure glucose syrup and purple sweet potato products.

After the curve was formed, the incremental area under the curve was calculated. The area under the horizontal basic line of the preprandial blood glucose level was not included [34] The area under the curve represented the total glycemic response of food products consumed as samples. The results of the calculation of the area under the curve of each respondent subject for each sample are shown in Figure 3.

Table 5 shows the averaged area under the curve of ten respondent subjects for each sample from the biggest to the smallest.

The result of one-way analysis of variance (ANOVA) revealed there were differences in the area under the curve of each product (*p* < 0.05) due to the different treatments in the processing of purple sweet potato products. The change in structures might affect the postprandial response to starchy food [35]. Processing had been proven to affect the area under the curve, therefore least significant difference test. LSD revealed no significant difference (*α* = 0.05) between purple sweet potato noodles and resistant starch-rich purple sweet potato noodles (Table 5). However, there was a significant difference between boiled purple sweet potatoes and pure glucose syrup. The lack of difference between purple sweet potato noodles and resistant starch-rich purple sweet potato noodles might be due to the disregard of available carbohydrates in the serving size. The serving size affected respondent subjects' time to consume the samples, over 15 minutes. The food products used as the sample should have been ingested within 10-15 minutes [34], to avoid them affecting the blood glucose level.

LSD also showed that the area under the curve of pure glucose syrup was the biggest, with a significant difference from purple sweet potato products. Pure glucose syrup had an average response of 5221.50 unit areas. Glucose syrup was selected as the control and comparison because of its pure glucose composition and how it could be absorbed immediately by the body into the metabolism system, giving glucose syrup 100 on the glycemic index value [36].

Among the purple sweet potato products, boiled purple sweet potatoes had the biggest area under the curve with 4317 unit areas. This was caused by how the starch in purple sweet potatoes had gelatinized completely in the boiling process at 100°C for 30 minutes. Purple sweet potato starch needs a temperature of 75-88°C for 36 minutes to gelatinize [37]. The starch granules in boiled purple sweet potatoes had gelatinized and distracted completely. The starch granule distraction leads the molecules to be easily hydrolyzed by enzymes [38]. This quick reaction from the enzymes resulted in the fast increment of blood glucose level. Thus, foods with complete starch gelatinization have high glycemic response [39].

Purple sweet potato noodles had 3279 unit area under the curve, smaller than boiled purple sweet potatoes but bigger than resistant starch-rich purple sweet potato noodles. Resistant starch-rich purple sweet potato noodles were the smallest with 3039 unit areas. This was due to the product's main ingredient being type 3 resistant starch (RS3) purple sweet potato flour that was made with partial gelatinization and retrogradation process. Partial gelatinization opened the amylose and amylopectin bond structures. Then, in the cooling process, compact starch molecules were restructured and stabilized with hydrogen bonds, creating starch that was difficult to hydrolyze by digestive enzymes [40]. Resistant starch-rich purple sweet potato noodles contained starch that was resistant to the acidity and enzymes in the small intestines so that the starch was able to reach the colon to be fermented, resulting in short-chain fatty acids such as acetate acid, propionate acid, and butyrate acid. These fatty acids would then be absorbed in the colon and metabolized to generate energy.

Processing starch to produce resistant starch is one way to lower glycemic response values. Resistant starch type 3, due to its special properties as a result of the retrogradation process, is not digested in the small intestine. However, after 2 hours, this type of resistant starch will be fermented in the colon resulting in short-chain fatty acids. This type of resistant starch has beneficial effects on recuperating insulin resistance and lowering blood glucose in type 2 diabetes mellitus patients. Blood glucose levels of respondent subjects after consuming these food products were also quite stable even after two hours. A smaller area under the curve indicated slower digestion of carbohydrates that resulted in slower and less glucose release, so the curve had gentler response and lower peak level of glycemic response, and vice versa.

Some respondent subjects only had a minimum difference in the area under the curve for different purple sweet potato products; some did not. Each respondent subject displayed different glycemic response curves with different peaks and glycemic index value indications. These varying results were assumed to be due to several factors of physiological responses such as metabolism, stress level, physiological condition, and fasting time.

#### 3.4.4. Glycemic Index Value Indication

**Figure 4:**
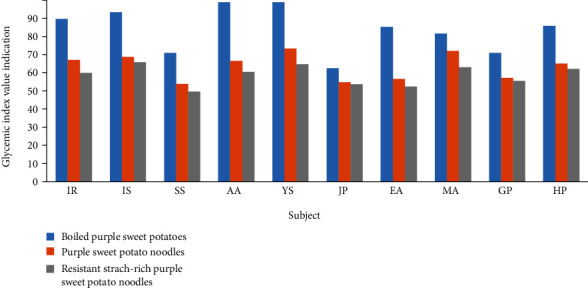
The glycemic index value indication of each respondent subject for purple sweet potato products.

The area under the curve that showcases glycemic response becomes the reference in calculating the glycemic index value of a food product. As applied in this research, the glycemic index value indications of purple sweet potato products were determined by dividing the AUC of each purple sweet potato product by the AUC of pure glucose syrup, then multiplying by 100. This equation came from the glycemic index equation; however, this research called these results shown in Figure 4 indication because available carbohydrates were not calculated.

Table 6 shows the averaged glycemic index value indications of all 10 respondent subjects for each purple sweet potato products, sorted from the highest to the lowest. The purple sweet potato product with the highest glycemic index value indication was boiled purple sweet potatoes with 83.75. Purple sweet potato noodles had 63.50, and resistant starch-rich purple sweet potato noodles had the lowest value with 58.74. It is suggested that the resistant starch content of the purple sweet potato product determined the value of glycemic index indication whereas the product containing higher resistant starch content (purple sweet potato noodles and resistant starch-rich purple sweet potato (Table 2)) had a lower glycemic index value indication (Table 6). Resistant starch lowers the glycemic index through reducing total starch that can be digested and delaying the rate of intestinal digestion by inhibiting *α*-amylase enzyme directly [10].

Based on general food glycemic index (GI) values, boiled purple sweet potatoes fell under food with high GI of over 70, while both noodles fell under the medium range with 55-70. Even though both noodles were in the medium range, resistant starch-rich purple sweet potato noodles still showed the lowest GI due to the main ingredient, RS3, that was made through partial gelatinization and retrogradation process and formed starch structures that were hard to digest. Different treatments and processes in making purple sweet potato products resulted in different glycemic index value indications.

## 4. Conclusion

The purple sweet potato products had different GI values depending on their processing method. The RSPN had the lowest glycemic index value if compared to BSP and SPN (58.7, 63.5, and 83.7, respectively) and fell under food with medium GI, but the differences were not statistically significant. The pattern of the GI value of the products was concomitant with the pattern of the rate of starch hydrolysis with *α*-amylase enzyme, and it was inversely proportional with their resistant starch content. Partial gelatinization of sweet potato flour has failed to reduce GI of noodles as indicated that RSPN and SPN were classified as medium GI food, but the process has successfully maintained the total phenolic and anthocyanin and increased resistant starch content of the noodle. Processing of purple sweet potato into noodles lowered the GI category, and when the flour was partially gelatinized and retrograded, the noodles had more potentiality as a functional food due to their high total phenolic and anthocyanin content.

## Figures and Tables

**Table 1:** Proximate composition.

Composition	Boiled purple sweet potato	Purple sweet potato noodles	Resistant starch-rich purple sweet potato noodles
Moisture (%wb)	62.83 ± 0.70^a^	53.02 ± 0.78^b^	44.13 ± 0.34^c^
Ash (%db)	2.99 ± 0.32^a^	2.55 ± 0.42^a^	1.38 ± 0.16^b^
Protein (%db)	3.22 ± 0.31^a^	2.38 ± 0.12^b^	1.70 ± 0.36^c^
Fat (%db)	1.01 ± 0.03^a^	0.78 ± 0.01^b^	0.63 ± 0.02^c^
Carbohydrate (%db)	92.78 ± 0.48^c^	94.29 ± 0.50^b^	96.29 ± 0.38^a^

Values are the mean ± standard deviation. Values in the same row followed by different letters are significantly different (*p* < 0.05).

**Table 2:** Total phenolic, anthocyanin, and resistant starch contents.

Content	Boiled purple sweet potato	Purple sweet potato noodles	Resistant starch-rich purple sweet potato noodles
Total Phenolic mg GAE/100 g)	357.33 ± 1.28^a^	195.58 ± 2.06^c^	327.10 ± 2.78^b^
Anthocyanin (mg/100 g)	95.64 ± 0.39^a^	44.71 ± 0.23^b^	93.94 ± 0.43^a^
Resistant starch (%)	2.99 ± 0.20^c^	6.52 ± 0.16^b^	14.29 ± 0.17^a^

Values are mean ± standard deviation. Values in the same row followed by different letters are significantly different (*p* < 0.05).

**Table 3:** The characteristics of respondent subjects.

No.	Subject	Sex	Age of years	Weight (kg)	Height (m)	BMI (kg/m^2^)	Preprandial glucose level (mg/dL)
1	IR	F	22	47	1.57	19.07	84.25
2	IS	F	21	50	1.60	19.53	86.50
3	SS	F	21	49	1.56	20.13	89.25
4	AA	F	22	51	1.60	19.92	87.00
5	YS	F	22	48	1.59	18.99	89.75
6	JP	F	22	55	1.58	22.03	82.00
7	EA	F	22	63	1.66	22.86	83.00
8	MA	M	25	66	1.70	22.84	85.75
9	GP	M	21	48	1.54	20.24	86.00
10	HP	M	22	58	1.60	22.66	88.75
	Mean	—	22	53.5	1.60	20.83	86.23

**Table 4:** Serving size of purple sweet potato products given to respondent subjects.

Product	Carbohydrate by difference (%wb)	Serving size/person (g)	Serving size/10 persons (g)
Boiled purple sweet potatoes	34.48	145.00	1450.03
Purple sweet potato noodles	44.30	112.88	1128.77
Resistant starch-rich purple sweet potato noodles	53.98	92.95	929.50

**Table 5:** The averaged area under the curve of all respondent subjects for pure glucose syrup and purple sweet potato products.

Product	AUC (unit area)
Pure glucose syrup	5221.50 ± 625.56^c^
Boiled purple sweet potatoes	4317.00 ± 390.76^b^
Purple sweet potato noodles	3279.00 ± 170.32^a^
Resistant starch-rich purple sweet potato noodles	3039.00 ± 152.18^a^

Values are the mean ± standard deviation. Values in the same column followed by different letters are significantly different (*p* < 0.05).

**Table 6:** The averaged glycemic index value indication of all respondent subjects for each purple sweet potato product.

Product	Glycemic index value indication
Boiled purple sweet potatoes	83.75 ± 12.34
Purple sweet potato noodles	63.50 ± 7.29
Resistant starch-rich purple sweet potato noodles	58.74 ± 5.55

## Data Availability

All data are included in the manuscript.
